# Design and validation of a community resilience scale in contexts of adversity

**DOI:** 10.3389/fpubh.2026.1850935

**Published:** 2026-08-19

**Authors:** Carolina Alzugaray, Alejandro Reyes-Reyes, Nekane Basabe, Anna Wlodarczyk, Loreto Villagrán, Larraitz Zumeta

**Affiliations:** 1 Escuela de Psicología, Facultad de Ciencias Sociales y Comunicaciones, Universidad Santo Tomás, Concepción, Chile; 2 Departamento de Psicología Social y Metodología de Ciencias del Comportamiento, Universidad del País Vasco, San Sebastián, Spain; 3 Escuela de Psicología, Universidad Católica del Norte, Antofagasta, Chile; 4 Departamento de Psicología, Universidad de Concepción, Concepción, Chile

**Keywords:** collective efficacy, community resilience, confirmatory factor analysis, psychometric, social capital

## Abstract

**Background:**

Community Resilience has drawn growing interest over recent decades. It is now a key variable in social research and in studies of stress and coping. Despite this, no consensus exists on its concept, components, or measurement.

**Objective:**

This study aimed to develop and examine the psychometric properties of a brief community resilience scale grounded in psychosocial processes among Spanish-speaking populations in Argentina and Chile. It also provides translations to support further validation and application in other contexts and presents a conceptual framework consistent with the measure.

**Methods:**

A convenience sample of 1,077 participants from Argentina (*n* = 724) and Chile (*n* = 353) was recruited. An initial set of items was subjected to exploratory factor analysis (EFA) to examine the underlying structure, followed by confirmatory factor analysis (CFA) to test competing models. A multigroup CFA was conducted to assess measurement invariance across countries. Reliability and validity evidence were examined through internal consistency and correlations with related constructs.

**Results:**

Community Resilience was defined as the capacity of a community to cope with collective adversity, regulate collective emotions, and use community resources and social capital to promote collective efficacy. Exploratory and Confirmatory factor analyses revealed that the CR scale comprises nine items in three dimensions: emotional regulation, social capital, and collective efficacy. The CR-9 scale showed satisfactory psychometric properties (*α* = 0.862; CFI = 0.981; RMSEA = 0.042). It proved to be a reliable measure with adequate convergent validity with communal mastery, subjective well-being, and social integration. It showed little correlation with individual resilience. Configural and metric invariance, as well as partial scalar invariance, were observed across countries (CFI = 0.961; SRMR = 0.048; RMSEA = 0.055).

**Conclusion:**

These findings provide initial support for a brief, multidimensional measure of community resilience (CR-9) among Spanish-speakers from Argentina and Chile. It measures three aspects: emotional regulation, social capital, and collective efficacy. The CR-9 performed well on reliability and validity tests in both groups. This scale is useful for community and social research, while its multilingual translations may facilitate future cross-cultural validation and use in diverse populations.

## Introduction

Collective adversities such as disasters caused by natural or technological threats, interpersonal violence, wars, violent social conflicts, and complex emergencies (1) often provoke negative effects on well-being and mental health (e.g., posttraumatic stress disorder) of the members of the affected community (2). Nevertheless, trauma literature suggests that about two-thirds of people exposed to stressful events reported positive personal and social-life changes (3, 4). Social relations and community interactions are important predictors of reparation and successful coping with disasters caused by natural and social threats (5–8).

### Individual and community perspectives on resilience

Individual resilience has been widely studied in the social sciences, constituting a well-established field of study (9–11). In general terms, resilience is defined as the ability to adapt and recover from disruptive events while maintaining essential functions and developing new strategies to face future challenges (12). It is understood as the ability to emerge unscathed from an adverse experience, to learn and grow from it (13), and to continue moving forward despite destabilizing events, difficult living conditions, and, at times, severe trauma (14).

In the face of disasters caused by natural and social threats, individuals are more likely to engage in collective coping strategies to restore and rebuild the affected community. A sense of community identity emerges from a shared fate, as people share problems, goals, perceptions of vulnerability, and social support (2).

Community Resilience has established itself as a key concept for understanding how communities cope with, withstand, and recover from adverse experiences that affect them collectively (15, 16). Various studies have shown that communities facing disasters develop resilience processes (17, 18). Patel and Gleason (19) define CR as “the ability to activate protective qualities and processes at the individual, community, institutional, and systems level to engage with hazards or stressors and cooperate with others to maintain or recover functionality and prosper while adapting to a new equilibrium and minimizing the accumulation of pre-existing or additional risks and vulnerabilities” (p. 162–163). This corresponds to the ways that people collectively organize, mobilize resources, and provide and expect solidarity and cohesion to overcome an emergency or a disaster, based on their shared social identity (15).

From an ecological perspective, CR can be understood as a multilevel process linking adaptive capacities across multiple levels, emerging from interactions among social, economic, and psychosocial systems (16, 20). Rather than residing solely in individuals, community resilience reflects the functioning of interdependent networks that enable the circulation of information, support, and resources (21). Consistent with this perspective, CR relies on social, organizational, and psychosocial capacities that help communities cope, adapt, and reorganize after crises (22). A key element is robust social networks, both formal and informal, which facilitate coordination, collective action, and shared decision-making, thereby enabling communities to address common problems more effectively (23, 24). Additionally, CR draws on community-based infrastructure and psychosocial factors, including hope, collective agency, and adaptability (25, 26), which, taken together, constitute the set of psychosocial processes necessary for a community to function resiliently.

From a measurement perspective, previous research has operationalized CR in diverse ways. The reviewed instruments for assessing CR exhibit variations in their factor structures, suggesting a lack of consensus on the construct’s conceptualization. For example, the Community Resilience Assessment Composite Measure (CCRAM) has been validated as a multidimensional scale comprising five factors: leadership, collective efficacy, preparedness, place attachment, and social trust (27). Similarly, Pfefferbaum et al. (28) validated an instrument composed of four dimensions: care, resources, transformative potential, and disaster management. Finally, Ruiz Pérez (29) proposed a two-dimensional scale linking CR to the dimensions of communal coping and collective efficacy. However, despite variations among the scales, their factor structures suggest the construct’s multidimensional nature.

### Community resilience: associated factors

Community Resilience goes beyond the mere ability to recover from disasters or collective hardships. It involves a network of resources, processes, and social relationships that support and enhance a community’s positive adaptation to adversity and is a multidimensional construct integrating shared emotional processes, collective perceptions of efficacy, social capital, and community well-being (30). Based on a comprehensive review of the literature, Alzugaray (30) proposes a theoretical model comprising three dimensions: social capital, collective efficacy, and emotional regulation.

### Emotional regulation (ER)

Emotional regulation is understood as the set of strategies individuals use to modulate their emotions, influencing their type, intensity, duration, and expression (31). This can be both extrinsic and intrinsic and involves conscious or unconscious strategies for managing emotional responses in various situations. These processes have significant implications for people’s psychological well-being and daily functioning (32).

Although the relationship between ER and CR has been little explored, evidence suggests that more resilient individuals and communities use strategies that evoke positive emotions to regulate negative emotional states (33). These emotions not only mitigate the effects of negative emotions but also expand attentional, cognitive, and behavioral resources, facilitating more adaptive responses to adversity (34). The social sharing of emotions in crisis contexts is associated with higher levels of social support, positive effects, and community cohesion in the short and long-term (35). Following a disaster, the collective expression of emotions and shared dialogue strengthen solidarity, empathy, and community identity, contributing to greater social resilience (36). Likewise, groups that actively manage their emotions through open-dialogue spaces achieve more cohesive and effective collective responses to adversity (37).

### Social capital (SC)

Putnam (38, p. 19) defines SC as “the characteristics of social organizations, such as trust, norms, and networks that can enhance the efficiency of society by facilitating coordinated action.” Traditionally, this concept refers to the resources and benefits that communities derive from their social relationships. In the post-pandemic era and the digital age, it has expanded to include new forms of participation and strengthened trust. These elements are crucial for community resilience (39). SC can serve as the framework for communities to rebuild after a disaster (40). Communities with high levels of SC are better organized and respond more effectively to adverse events, a result of the trust and reciprocity among their members (41–43).

Evidence consistently shows that SC is a key factor in CR to disasters. Strong social networks, based on trust, reciprocity, social cohesion, and civic participation, facilitate the mobilization of resources, mutual support, and the coordination of collective responses, while reducing fear and strengthening collective agency and self-efficacy (44, 45). Likewise, communities with robust social capital demonstrate greater capacities for preparedness, reconstruction, and adaptation, as well as lower resource losses following socio-natural events (46, 47). In contexts of high structural vulnerability, such as Latin American communities affected by violence and marginalization, practices of solidarity, cohesion, and community organization are particularly relevant for sustaining resilient processes (26, 48).

### Collective efficacy (CE)

Collective efficacy is defined as the shared belief within a community that it can organize and take action to achieve desired outcomes (22). This belief, in turn, influences how communities approach environmental challenges by fostering strategic and optimistic action toward shared goals (49). As a result, collective efficacy also impacts preparedness and response to adversity by strengthening communities’ ability to organize, coordinate actions, and mobilize shared resources (50).

Community Resilience is strengthened through organizational capacity, communication, and social support, which cushion the negative effects of traumatic events and sustain social, economic, and environmental functioning in the face of disruptive impacts (51–53). Importantly, these capacities are supported by cultural knowledge, social resources, and organizational strategies that enable challenges to be addressed effectively (26). Building on this, collective efficacy and community resilience together foster greater connection with the environment, thereby increasing community participation and engagement (54).

Empirical evidence indicates a significant relationship between CE and CR across contexts of adversity. In natural disaster scenarios, such as hurricanes and floods (55–57), as well as in contexts of criminal violence in Colombia (29), communities with higher CE demonstrate better response capabilities, lower psychosocial impacts, and greater collective action. In turn, CR strengthens CE through cohesion, social learning, and active participation, thereby consolidating community recovery and adaptation.

In sum, in the present study, CR is theoretically conceptualized as a higher-order construct integrating three core dimensions—emotional regulation, social capital, and collective efficacy. These dimensions are understood as interrelated psychosocial processes that jointly contribute to the community’s capacity to cope with, adapt to, and recover from collective adversity (30). This conceptualization is consistent with systemic and ecological perspectives of resilience, which emphasize the interaction of multiple resources and processes across levels (6, 12).

### Community resilience and its psychosocial correlates

Beyond its internal structure, CR has been associated with theoretically related psychosocial constructs, including subjective well-being, social integration, and communal mastery (58). Empirical evidence suggests that adverse collective experiences tend to mobilize social networks, leading people to seek social support from family and friends and to adopt communal coping strategies (8). These adverse experiences are correlated with all social well-being facets -social integration, contribution, acceptance, actualization, and coherence (59, 60). In the same sense, the organizational capacity of communities, communication, and social support are factors that may mitigate the negative effects of the traumatic events (61, 62).

Closely related to social integration is the concept of communal mastery, which is associated with prosocial coping strategies, such as problem-solving with others and using social support from friends, family, and significant others (63, 64). This concept refers to the individual’s perception that life challenges are better managed by collective action (63). The activation of social networks, the perception that challenges can be addressed collectively, and the belief in shared agency constitute interrelated psychosocial processes that buffer the negative effects of collective trauma and promote adaptive community functioning (61, 62, 64).

The literature indicates that CR refers to a group’s or community’s ability to face adversity, maintain social connectedness, and construct shared meaning in response to challenges. As such, CR is rooted in communal orientations that influence both individual and social well-being (65). In this context, interest in CR among social scientists has grown (66, 67), highlighting the need for stress and coping researchers to clarify its definition further and develop tools to measure it across situations in which groups experience disadvantage.

However, most psychometric instruments available for measuring CR have been designed for specific socio-natural disaster contexts (6, 27), neglecting other forms of psychosocial adversity, such as social exclusion or structural violence. The development of a scale that accounts for these factors will enable a more comprehensive assessment applicable across contexts of collective adversity. Furthermore, traditional approaches to measuring CR have focused primarily on structural indicators, such as the availability of material resources or the speed of recovery following a disaster, and do not consider psychosocial dimensions that influence a community’s ability to overcome adverse conditions, such as social cohesion and support, perceptions of collective self-efficacy, or emotional regulation (19, 68), key determinants of community resilience.

Community resilience is crucial for community adaptation to adversity, so an appropriate assessment scale is needed. Assessing CR helps explain collective adaptation and recovery, identifies protective group-level factors, and guides effective interventions for vulnerable communities. This study aimed to develop a community resilience scale and establish its psychometric properties in Spanish-speaking populations in Argentina and Chile. Additionally, the scale is translated into several languages to facilitate validation studies and broader use.

## Materials and methods

### Scale development

To develop the scale, a systematic review of empirical studies published in the social sciences (Web of Science, Scopus, and PsycINFO) was conducted using the keywords “resilience,” “community resilience,” and “collective resilience.” Studies that included quantitative measures of community resilience were selected, resulting in eight instruments (6, 27, 68–73).

In the next step, two independent expert judges evaluated 23 attributes of community resilience and classified them into three dimensions: emotional regulation (ER), social capital (SC), and collective efficacy (CE). Inter-coder reliability was satisfactory (Krippendorff’s *α* = 0.866; 2 coders, 23 observation units).

Based on this process, a preliminary 51-item scale was developed, from which a 12-item short version was subsequently derived.

To develop the short form, the initial 51-item scale Alzugaray (30) was used as the starting point. Both empirical and theoretical criteria guided item selection. First, exploratory factor analysis (EFA) was used to retain items with factor loadings ≥ 0.50. To ensure adequate representation of each latent dimension, four items per factor were selected, a criterion supported in the literature (74, 75). When multiple items met these criteria, a panel of three expert judges evaluated each item’s representativeness and selected the most theoretically relevant. Inter-rater agreement was satisfactory (Aiken’s V > 0.70). This process resulted in a 12-item short version of the scale.

To facilitate future cross-cultural applications, the original Spanish version of the scale was translated into English and Portuguese by bilingual collaborators familiar with psychosocial terminology. The research team reviewed both versions to ensure semantic and conceptual equivalence with the original items, particularly across the three theoretical dimensions of emotional regulation, social capital, and collective efficacy. Discrepancies were resolved by consensus, and the translated versions are provided in the Supplementary materials.

### Study design

An instrumental study design (76) was used to develop and establish the scale’s psychometric properties.

### Participants

Using non-probabilistic convenience sampling, a sample of 1,077 adults was selected, comprising Argentines (*n* = 724) and Chileans (*n* = 353), of all genders (56.3% women, 43.7% men), aged between 18 and 67 years (*M* = 24, *SD* = 6.22). Most participants were students and members of the general population in both Argentina (59.8% women, *M* = 22.38, *SD* = 4.45) and Chile (49.1% women, *M* = 27.94, *SD* = 7.91). The adversities reported by participants consisted of 46.1% general and political violence (e.g., theft, intimate partner violence, abuse by law enforcement), 15.7% disasters caused by natural hazards (e.g., floods, earthquakes, volcanic eruptions), 14.4% to economic crises, 14.4% to disasters caused by human behavior (e.g., fires and pollution), and 15.6% to other causes (e.g., illnesses and accidents). The communities chosen to cope with these adversities were the neighborhood (48.2%), the city (30%), the municipality (15.6%), and others such as family and friends (5.9%).

### Measurement

*Community resilience scale* (CR-12) (30), a 12-item Likert scale with five response options, ranging from 1 = strongly disagree to 5 = strongly agree. The scale measures perceptions of community capacities and relationships, represented in three dimensions of community resilience: Emotional Regulation (RE, items 4, 5, 9 and 10), Social Capital (SC, items 2, 3, 11 and 12) and Collective Efficacy (EC, items 1, 6, 7 and 8) (see Appendix A).

*Individual resilience* (IR, 77), (Maltby et al., 93) a 12-item Likert scale with five response options, ranging from 1 = strongly disagree to 5 = strongly agree. The scale measures resilience as a trait, derived from three common mechanisms identified in ecological theory: engineering resilience (4 items), ecological resilience (4 items), and adaptive resilience (4 items). The measure demonstrated good reliability in the sample: *α* = 0.871.

*Communal mastery scale* (CM; 63), (Hervás et al., 94)an 8-item Likert-type scale with four response levels, ranging from 1 = strongly disagree to 4 = strongly agree. It measures the extent to which individuals perceive themselves as capable of effectively achieving their goals and coping with life’s challenges thanks to their connections with significant others. The estimated reliability coefficient in the sample was acceptable: *α* = 0.729.

*Subjective well-being* (RWB-PHI, 78), an 11-item scale with 10 response levels ranging from 0 = strongly disagree to 10 = strongly agree. The scale measures four domains of recalled well-being: General (2 items), eudemonic (6 items), hedonic (2 items), and social (1 item). The internal reliability index was acceptable: *α* = 0.875.

*Social integration* (SI, 88); adapted by Bobowik et al. (59). We used 3 items to assess social integration (e.g., I feel close to other people in my community). Participants were asked to rate their level of agreement with each statement on a 5-point scale, ranging from 1 = strongly disagree to 5 = strongly agree. The internal reliability index was acceptable: *α* = 0.708.

*Socio-demographic survey*. A brief survey that asked about nationality, age, gender, the adversity or situation to which they were exposed, and the group they considered their community.

### Procedures

The questionnaires were administered using a combination of methods: an online survey via Survey Monkey and an in-person survey conducted in students’ classrooms. Prior to completing the questionnaires, participants signed an informed consent form that guaranteed their autonomy, minimal risk of exposure, anonymity, confidentiality, and compliance with the Personal Data Protection Act. The study adhered to the ethical principles of the World Medical Association’s Declaration of Helsinki (77).

### Data analysis

Descriptive statistics, alpha reliability coefficients, and correlation indices were estimated. Additionally, exploratory factor analysis (EFA) and confirmatory factor analysis (CFA) were conducted. To examine and cross-validate the factorial structure of the CR scale, the total sample (*n* = 1,077) was randomly divided into two independent subsamples. The first subsample (*n* = 522) was used for exploratory factor analysis, allowing the scale’s empirical dimensionality to be examined without imposing a prior structure. The EFA followed an iterative process of model refinement, combining statistical criteria (factor loadings and cross-loadings) and theoretical considerations. Based on the exploratory results and the theoretical model of community resilience, three competing factorial models were specified. These models were subsequently tested in the second subsample (*n* = 555) using confirmatory factor analysis. The final model was selected according to goodness-of-fit indices, parsimony, and theoretical interpretability. The estimation procedure applied was maximum likelihood with robust estimation (78). In addition to the chi-square test, the following indices were used to assess model fit: the CFI (Comparative Fit Index) and the TLI (Tucker-Lewis Index), for which values above 0.90 are considered acceptable, as well as the SRMR (Standardized Root Mean Square Residual), for which values below or close to 0.08 indicate a relatively good model fit (79). To assess the factorial invariance of the instrument across countries (Argentina and Chile), a multigroup confirmatory factor analysis (MG-CFA) was performed using a hierarchical progressive restriction procedure. Four nested models—configural, metric, scalar, and strict—were compared, and the change in fit indices between successive models was evaluated. Following the criteria of Cheung and Rensvold (80) and Chen (81), two models were considered equivalent when ΔCFI ≤ 0.01, complemented by ΔRMSEA ≤ 0.015 and ΔSRMR ≤ 0.030 for metric invariance, and ΔSRMR ≤ 0.010 for scalar invariance. This approach allows us to determine whether the observed differences between groups reflect true differences in the construct or measurement biases attributable to the instrument’s structure. Furthermore, we include the Akaike Information Criterion (AIC), with lower values indicating a better, more parsimonious model (82). The figure showing the result presents the standardized solution. This procedure provided evidence that the CR scale is best represented by a multidimensional structure comprising emotional regulation, social capital, and collective efficacy. Descriptive statistics, bivariate correlations, and internal consistency coefficients were computed using IBM SPSS Statistics v.27. Exploratory factor analyses (EFA) were conducted using JASP v 0.96, whereas confirmatory factor analyses (CFA) and measurement invariance analyses were performed using Mplus v. 7.1 (83).

## Results

### Descriptive statistics and reliability estimates

Table 1 presents descriptive statistics, internal consistency coefficients, and bivariate correlations among the study variables. The mean scores were generally near the midpoints of their respective scales, although Collective Efficacy had a slightly lower mean. The total Community Resilience score was strongly and positively correlated with its three dimensions: Emotional Regulation, Social Capital, and Collective Efficacy. In addition, Community Resilience showed positive, low-to-moderate correlations with Individual Resilience, Communal Mastery, Subjective Well-being, and Social Integration. Regarding reliability, the Community Resilience scale showed good internal consistency (*α* = 0.862). The Emotional Regulation (*α* = 0.709), Social Capital (*α* = 0.692), and Collective Efficacy (*α* = 0.807) dimensions showed acceptable to good internal consistency.

**Table 1:** Descriptive statistics, internal consistency, and bivariate correlations among study variables.

Variables	M(SD)	α	1.	2.	3.	4.	5.	6.	7.	8.
1. CR	27.30 (6,35)	0.862	—							
2. ER	9.53 (2.31)	0.709	0.89^**^	—						
3. SC	9.06 (2.41)	0.692	0.88^**^	0.74^**^	—					
4. CE	8.74 (2.59)	0.807	0.85^**^	0.62^**^	0.56^**^	—				
5. Individual resilience	43.04 (7.63)	0.871	0.26^**^	0.25^**^	0.19^**^	0.25**	—			
6. Communal mastery	29.31 (4.01)	0.729	0.21^**^	0.19^**^	0.20^**^	0.15**	0.32**	—		
7. PHI	82.29(16.78)	0.875	0.16^**^	0.16^**^	0.12^**^	0.14**	0.27**	0.26**	—	
8. Social integration	18.09 (3.24)	0.708	0.35^*^	0.34^**^	0.31^**^	0.26^**^	0.19^**^	0.34**	0.18^**^	—

CR, Community resilience; ER, Emotional regulation; SC, Social capital; CE, Collective efficacy; PHI, Subjective well-being; total *N* = 1,077 *, *p* < 0.05 **, *p* < 0.01.

### Exploratory factor analysis

Three exploratory factor analyses (EFAs) were conducted using direct Oblimin oblique rotation to examine the instrument’s underlying factor structure. The minimum error method was employed, and the number of factors to retain was determined based on the results of the parallel analysis.

The first EFA included all 12 items of the scale. The indices of model fit supported the appropriateness of factor analysis (KMO = 0.91; Bartlett’s sphericity test: *χ*^2^ = 2160.99, df = 66, *p* < 0.001). The initial solution suggested a four-factor structure, which explained 47.7% of the total variance. All items had factor loadings greater than 0.30; however, item 10 showed significant cross-loadings exceeding 0.30. Item 9 was excluded from further analyses because it demonstrated low factorial specificity; specifically, it did not have any salient loading in the pattern matrix, indicating it could not be clearly assigned to any specific factor. Its associations in the structure matrix appeared to reflect only shared variance among correlated factors, rather than belonging to a coherent factor. The correlations between the factors ranged from 0.10 to 0.69.

Considering these results, a second EFA was estimated excluding the fourth factor, since it was defined solely by item 10. Furthermore, this item had a factor loading of 0.494 on the third factor, which justified its retention in the alternative solution. This second structure consisted of 11 items distributed across three factors: Factor 1, comprising items 6, 7, 8, and 12; Factor 2, comprising items 1, 2, 3, and 11; and Factor 3, comprising items 4, 5, and 10.

Subsequently, a conceptual review of the factor composition was conducted. As a result, items 1 and 12 were eliminated because their content did not present a sufficiently clear theoretical correspondence with the factors into which they were grouped. Thus, a final exploratory factor structure with three factors was obtained, each comprising three items.

### Confirmatory factorial analysis and invariance

**Figure 1:**
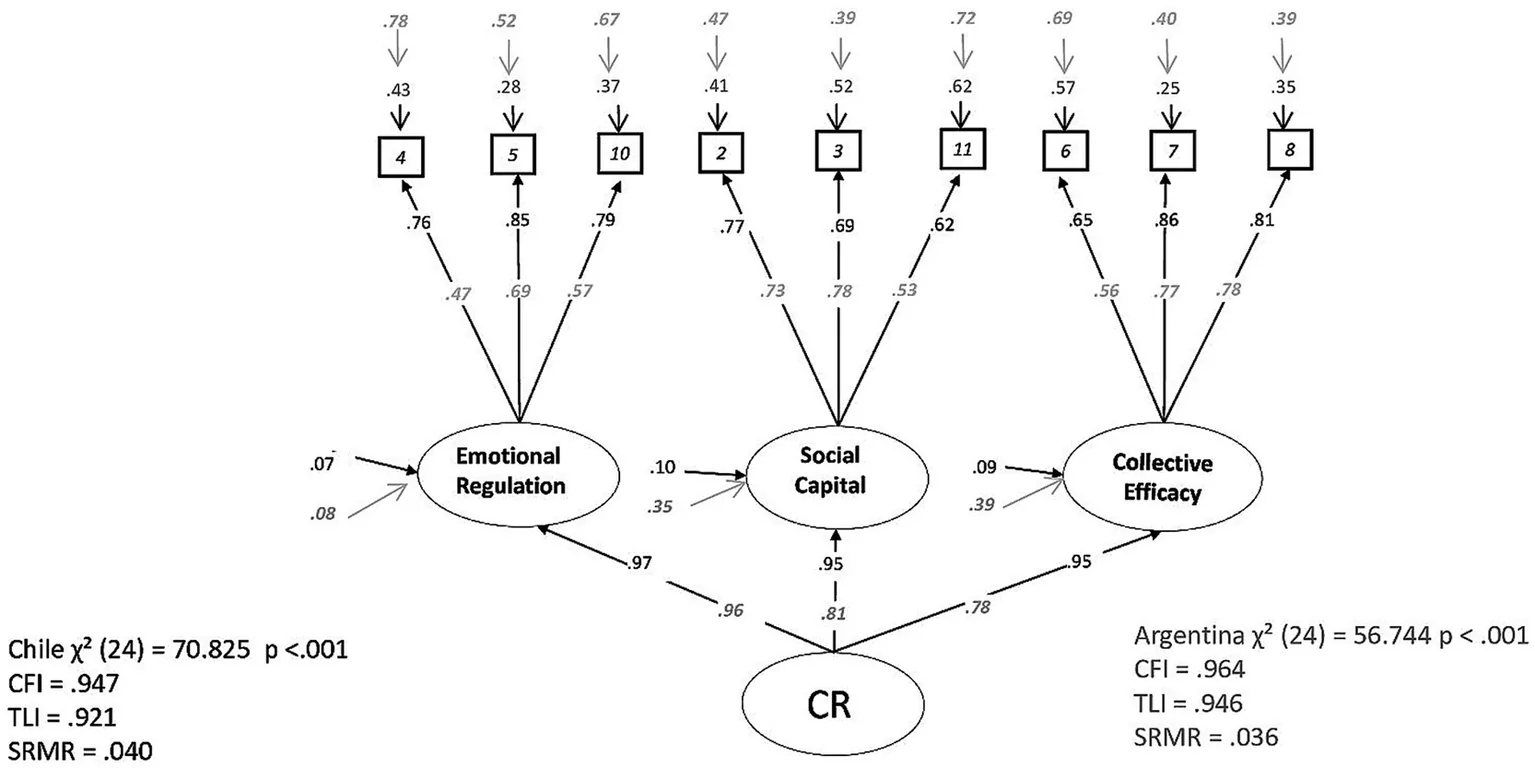
Second order CFA.

When comparing the confirmatory factor models, Model 2 exhibited the best overall fit indices. This model showed an adequate *χ*^2^/df ratio (*χ*^2^/df = 1.99), along with high CFI (0.981) and TLI (0.972) values, all of which exceeded the recommended cutoff of 0.95. It also exhibited low residual indices, with SRMR = 0.028 and RMSEA = 0.042, whose 90% confidence interval ranged from 0.027 to 0.057, suggesting a good fit of the model to the data. Furthermore, this model had the lowest AIC value (12,450), supporting its relative superiority over the alternative models. Consequently, Model 2 was selected as the most parsimonious and empirically appropriate factorial solution (see Table 2, Figure 1).

**Table 2:** Fit indices for alternative confirmatory factor analysis model.

Model	*χ* ^2^	gl	*p*	*χ*^2^/gl	CFI	TLI	RMSEA	IC 90% RMSEA	SRMR	AIC
Model 1	189.801	54	< 0.001	3.51	0.926	0.910	0.067	0.059, 0.076	0.044	16,520
Model 2	47.795	24	0.003	1.99	0.981	0.972	0.042	0.027, 0.057	0.028	12,450
Model 3	127.250	51	< 0.001	2,49	0.959	0.946	0.052	0.043, 0.061	0.036	16,430

Model 1: one-factor model with 12 items. Model 2 = higher-order model with three first-order factors and nine items, with three items per factor. Model 3 = original higher-order model with three first-order factors and 12 items, with four items per factor. CFI, Comparative fit index; TLI, Tucker-lewis index; RMSEA, Root mean square error of approximation; SRMR, Standardized root mean square residual; AIC, Akaike information criterion.

Table 3 presents the measurement invariance analysis of the nine-item, three-factor Community Resilience scale across the Argentinean and Chilean samples. Measurement invariance was tested sequentially by estimating configural, metric, scalar, and strict invariance models. The configural model showed an acceptable fit to the data, supporting the same factorial structure across countries. The metric invariance model also showed an adequate fit, with changes in fit indices below the recommended cutoff criteria (ΔCFI < 0.010 and ΔRMSEA < 0.015), indicating that factor loadings were equivalent across groups. However, the full scalar invariance model showed a decrease in model fit, particularly in CFI, suggesting that the equality constraints on all item intercepts were not fully supported. Therefore, a partial scalar-invariance model was estimated by releasing the constraints on the non-invariant intercepts. This model showed an acceptable fit, with changes in CFI and RMSEA within the recommended thresholds (ΔCFI = 0.006 and ΔRMSEA = 0.002), supporting partial scalar invariance across countries. These findings indicate that the CR-9 scale presents configural and metric invariance, as well as partial scalar invariance, allowing meaningful comparisons of latent associations across Argentina and Chile. However, comparisons of latent means should be interpreted with caution.

**Table 3:** Measurement invariance of the CR-9 scale across Argentina and Chile.

Invariance Model	*χ* ^2^	Df	AIC	CFI	TLI	SRMR	RMSEA	CI 90% RMSEA	∆CFI	∆RMSEA
1. Together	47.795	24	12,450	0.981	0.972	0.028	0.042	0.027 0.057	-	-
2. Argentina	56.744	24	8,300	0.964	0.946	0.036	0.0650	0.040, 0.081		
3. Chile	70.825	24	3,981	0.947	0.921	0.040	0.104	0.077 0.133		
1. Configural	85.136	44	12,290	0.965	0.943	0.037	0.058	0.043, 0.073	-	-
2. Metric	102.623	56	12,270	0.961	0.950	0.048	0.055	0.040, 0.069	0.004	0.003
Scalar	134.080	61	12,300	0.939	0.928	0.059	0.066	0.053, 0.079	0.022	0.011
Partial scalar invariance	113.800	59	12,278	0.955	0.946	0.051	0.057	0.043,071	0.006	0.002
Strict	166.781	70	12,330	0.919	0.916	0.086	0.071	0.059, 0.083	0.020	0.005
Structural	267.944	78	12,450	0.840	0.853	0.150	0.094	0.083, 0.105	0.079	0.023

*χ*^2^, Chi-Square; df, degrees of freedom; CFI, Comparative fit index; SRMR, Standardized root mean square residual; RMSEA, Root mean square error of approximation and 90% confidence interval. All *χ*^2^: *p* < 0.001.

### Discriminant and convergent validity

Evidence based on relationships with other variables supported the construct validity of the CR-9 scale. As expected, the total Community Resilience score was strongly and positively associated with its three dimensions: Emotional Regulation (*r* = 0.89, *p* < 0.01), Social Capital (*r* = 0.88, *p* < 0.01), and Collective Efficacy (*r* = 0.85, *p* < 0.01), indicating strong internal coherence among the components of the construct. In addition, Community Resilience showed positive and statistically significant associations with theoretically related psychosocial variables, including Individual Resilience (*r* = 0.26, *p* < 0.01), Communal Mastery (*r* = 0.21, *p* < 0.01), Subjective Well-being (*r* = 0.16, *p* < 0.01), and Social Integration (*r* = 0.35, *p* < 0.05). These findings provide evidence of convergent validity, as higher perceived community resilience was associated with higher levels of individual and collective psychosocial resources. At the same time, the low-to-moderate magnitude of these correlations suggests that CR is related to, but not redundant with, individual resilience, communal mastery, subjective well-being, or social integration, providing preliminary evidence of discriminant validity (See Table 1).

## Discussion

This study examined CR in contexts of collective adversity and contributes to the literature by developing and providing initial validation of a brief multidimensional scale grounded in psychosocial processes.

The findings support a three-factor structure comprising emotional regulation, social capital, and collective efficacy, each represented by three items and integrated into a higher-order model of community resilience.

The initial exploratory analyses suggested a more complex four-factor configuration; however, item-level inspection and theoretical refinement supported a more parsimonious and conceptually meaningful three-factor, nine-item solution. Confirmatory factor analysis showed that, among the tested models, this structure achieved the best overall fit, supporting its adequacy and interpretability.

Together with the CFA results, the moderate-to-high intercorrelations among the three dimensions support conceptualizing community resilience as a higher-order construct. Rather than representing independent domains, emotional regulation, social capital, and collective efficacy appear to reflect interrelated psychosocial processes that converge into a broader latent construct. This interpretation is consistent with socioecological approaches, which conceptualize resilience as an emergent property arising from the interaction of multiple psychosocial processes.

At the dimensional level, the findings highlight emotional regulation as a core component of CR, suggesting that adaptive management of collective emotion is associated with more effective coping strategies in contexts of adversity (2, 8). Importantly, in this context, emotional regulation should not be understood solely as an individual-level process but as a collective phenomenon that emerges through social interaction. In situations of adversity, emotions are shared, communicated, and co-regulated within groups, giving rise to collective emotional regulation processes (2, 84). Furthermore, positive collective emotions—such as hope and shared confidence—may broaden cognitive and behavioral repertoires, facilitating collective action and strengthening collective efficacy (1, 34, 60, 85).

The second dimension, social capital, emerges as another component of CR. Consistent with prior literature, networks of trust, reciprocity, and cooperation appear to facilitate coordinated collective responses to adversity (26, 44, 86, 87). These resources are likely to support both social cohesion and the restoration of community functioning following disruptive events (19, 68, 88). It is important to acknowledge that social capital may not be evenly distributed within communities and may exclude marginalized groups. This suggests that the benefits of social capital for community resilience may depend on the inclusiveness and accessibility of social networks, especially in contexts characterized by structural vulnerability (89).

The identification of collective efficacy as a core dimension of CR aligns with previous research linking this construct to preparedness, coordinated crisis response, reduced psychosocial impacts, and community participation (45, 56). When communities perceive themselves as capable of acting together, they are more likely to mobilize resources, coordinate efforts, and sustain collective action, all of which are key processes in community adaptation to adversity (26, 44, 85, 87).

Furthermore, the analysis of measurement invariance across Argentina and Chile evidenced configural and metric invariance, indicating that the factorial structure and the relationships between items and latent dimensions are comparable across both contexts. However, scalar invariance was not supported, suggesting that comparisons of mean levels of community resilience between countries should be interpreted with caution, given that the sample’s relatively homogeneous composition (mostly students) may have contributed to the observed equivalence. Future research should examine the invariance of the measurement in more diverse samples to assess the generalizability of these results.

Taken together, these findings support the scale’s cross-contextual applicability at the structural level while highlighting potential cultural differences in response patterns.

An additional consideration is the diversity of adversities reported by participants, which may involve different psychosocial demands. While this variability supports the broad applicability of community resilience, it may also contribute to greater complexity in the observed patterns. These patterns do not operate uniformly across contexts of adversity. Different types of collective adversity may emphasize distinct psychosocial processes—for example, disasters tend to activate cohesion and coordination, while contexts of violence highlight social identity and organization. However, across contexts, collective adversities consistently mobilize shared processes such as social support, collective identity, and coordinated action that sustain community responses (6, 17). Accordingly, the findings should be interpreted as reflecting general trends rather than context-specific dynamics. Future research should examine the scale within more specific contexts to further assess its structural consistency.

Beyond its factorial structure, the study also examined the associations between community resilience and relevant psychosocial variables. The results showed positive and significant correlations between CR and community mastery, subjective well-being, and social integration, although the correlations were generally low to moderate.

Regarding communal mastery, its positive and significant association with community resilience reinforces the importance of perceiving challenges as more effectively addressed through collective action. This construct reflects a relational orientation to coping, characterized by the use of prosocial strategies, shared problem-solving, and reliance on social support networks. In this sense, communal mastery appears to contribute to adaptive community functioning by strengthening collective action and reinforcing shared beliefs about the community’s capacity to organize and respond to adversity. These findings are consistent with previous work highlighting the role of communal coping processes in shaping how communities mobilize resources and coordinate responses in the face of stressors (63).

The strongest association was observed with social integration, suggesting that perceived connectedness, sense of belonging, and active participation within the community play a central role in collective coping processes and in buffering the negative effects of adversity. These findings are consistent with prior research indicating that social embeddedness facilitates the circulation of support, information, and coordinated action, thereby enhancing community-level adaptive responses (37, 61).

In addition, community resilience showed a positive, although modest, association with subjective well-being. This relationship may reflect the role of shared emotional regulation, collective coping processes, and the construction of shared meanings in promoting both hedonic and eudaimonic aspects of well-being. In contexts of adversity, the collective processing of emotions and experiences can foster positive affect, reinforce a sense of purpose, and support psychological adjustment at both individual and collective levels (31, 60).

Another important finding is the low correlation observed between CR and IR. Although both constructs are fundamental for coping with adversity and are interrelated, theoretical and empirical evidence clearly distinguish between these levels of analysis (90, 91). In particular, previous studies suggest that individual-level changes do not necessarily translate into community-level transformations in response to adverse experiences. These findings suggest that community resilience cannot be understood as a mere aggregation of individual capacities, but rather as an emergent process grounded in relational and collective dynamics operating at a higher-order level (92).

Overall, the observed pattern of associations supports the multidimensional and distinct nature of community resilience while also highlighting its embeddedness within broader psychosocial systems. Additionally, these findings support the construct’s convergent validity.

Taken together, these findings indicate that community resilience is multidimensional—it allows communities to cope with adversity, manage shared emotions, mobilize social capital, and build collective efficacy. By providing initial evidence of reliability and validity, this study contributes both theory and practice, advancing research and intervention on CR, especially in contexts of collective disadvantage and social vulnerability.

## Limitations

This study analyzed CR in samples experiencing multiple collective disasters and traumas using a cross-sectional, retrospective design. An important limitation concerns the sampling procedure and the sample composition. Participants were recruited using a non-probabilistic convenience sampling strategy, which limits the representativeness of the findings and restricts their generalizability to broader community populations. In addition, the sample included a relatively high proportion of students and young adults, which may have influenced the observed patterns of CR, particularly given that age, educational context, and exposure to specific forms of collective adversity may shape how individuals perceive and mobilize community resources. Therefore, the results should be interpreted as providing evidence about community resilience within the specific samples and contexts examined, rather than as estimates that can be directly extrapolated to the general population. Further longitudinal research is required to investigate the mechanisms underlying CR effects in groups and individuals. Future studies should also use probabilistic or stratified sampling procedures and include more heterogeneous community samples across age, educational level, socioeconomic status, and territorial background. In addition, future research should explore relationships among CR, communal ways of coping, and Collective Posttraumatic Growth. Community resilience should also be investigated in other contexts, such as social exclusion, which particularly affects adolescents and young people. Another aspect to consider is the diversity of adverse situations that a person belonging to a community may experience, which may vary from one country to another, from one community to another, and over time. Such variability may shape the expression of community resilience, thereby modifying the factor structure and the invariance between the groups to be compared; therefore, additional studies are needed to examine these context-specific patterns.

## Conclusion

This study presents a preliminary psychometric validation of a CR Scale that supports the existence of three dimensions of CR: Emotional Regulation, Social Capital, and Collective Efficacy. Additionally, it provides evidence of the scale’s internal consistency and construct validity. Finally, community resilience was positively associated with subjective well-being and social integration, supporting its embeddedness in broader psychosocial and community processes. These findings provide initial evidence supporting the multidimensional nature of community resilience and offer a useful basis for future research and intervention in this field.

## Data Availability

The datasets presented in this study can be found in online repositories. The names of the repository/repositories and accession number(s) can be found at: https://osf.io/d8w6n/overview?view_only=034b8d58b78d48f8aa0ea6b93aeb08a9.
