# Supplementary material for: Design and validation of a community resilience scale in contexts of adversity
Source: Front Public Health. 2026 Aug 19;14:1850935. doi: 10.3389/fpubh.2026.1850935 (PMC13537183; doi:10.3389/fpubh.2026.1850935)
Supplement: Supplementary file 1 [file Supplementary_file_1.docx]

# Community Resilience Scale RC-12

(Alzugaray et al., 2018)

Please indicate a stressful or challenging event that has affected you

Natural disaster (e.g., flood, earthquake, volcanic eruption).

Disaster caused by human activity (e.g., fire, environmental pollution).

Political events.

Violence.

Economic crisis.

Other (please specify): _____________________________

Please indicate which of the following groups you consider to be your community in relation to the event described above

a) Neighborhood.

b) City.

c) Municipality / Local authority.

d) Other (please specify): _____________________________

Below are a number of statements. Please indicate the extent to which you agree or disagree with each statement

1 = Strongly disagree

2 = Disagree

3 = Neither agree nor disagree

4 = Agree

5 = Strongly agree

1. In situations of adversity, each member of our community clearly understands what actions they are expected to take.

2. In situations of adversity, members of our community communicate with leaders who are able to contribute to community improvement.

3. Our community collaborates with organizations and agencies outside the community to obtain necessary resources during adverse situations.

4. During adverse situations, we are able to manage negative emotions such as sadness and anger.

5. During adverse circumstances, we remain attentive to the needs of members of our community.

6. Our community is capable of addressing adverse situations independently, prior to receiving external assistance.

7. During adversity, our community organizes itself and plans actions before taking action.

8. Our community develops and implements the necessary skills to overcome adversity and achieve collective goals.

9. In our community, we do not become discouraged when faced with adversity.

10. When problems or adversities arise, members of my community come together and organize to provide support.

11. We have access to municipal (city or local authority) goods and services during adverse situations.

12. During adversity, members of our community regulate individuals who behave inappropriately or deviate from established norms.
