# Supplementary material for: Design and validation of a community resilience scale in contexts of adversity
Source: Front Public Health. 2026 Aug 19;14:1850935. doi: 10.3389/fpubh.2026.1850935 (PMC13537183; doi:10.3389/fpubh.2026.1850935)
Supplement: Supplementary file 2 [file Supplementary_file_2.docx]

### 1 Escala Resiliencia Comunitaria RC-12 and RC-9 (Alzugaray et al., 2018/2026)

**Señala un hecho estresante o de dificultad que te haya impactado**

a) Catástrofe natural (p. ej., inundación, terremoto, erupción).
b) Catástrofe generada por comportamiento humano (p. ej., incendio, contaminación).
c) Hechos políticos.
d) Violencia.
e) Crisis económica.
f) Otro (especificar): ______________________________________________________________

**Marca cuál de los siguientes grupos consideras como tu comunidad ante el hecho anteriormente señalado**

a) Barrio.

b) Ciudad.

c) Comuna

d) Otro (especificar)_________________________________________________________________

**A continuación, por favor, responda las siguientes preguntas, indicando el grado de acuerdo que tiene con cada una de las siguientes afirmaciones.**

| *Fuertemente en desacuerdo* | *En desacuerdo* | *Ni en desacuerdo ni en acuerdo* | *De acuerdo* | | *Fuertemente de acuerdo* | | |  |
| --- | --- | --- | --- | --- | --- | --- | --- | --- |
| 1 | 2 | 3 | 4 | | 5 | | |  |
| 1- En situaciones de adversidad, cada miembro de nuestra comunidad tiene claro lo que tiene que hacer. | | | | 1 | 2 | 3 | 4 | 5 |
| 2- En situaciones de adversidad, las personas en nuestra comunidad se comunican con los líderes que pueden ayudar a mejorar la comunidad | | | | 1 | 2 | 3 | 4 | 5 |
| 3- Nuestra comunidad trabaja con organizaciones y agencias fuera de la comunidad para obtener lo que necesita durante una adversidad. | | | | 1 | 2 | 3 | 4 | 5 |
| 4- Durante una adversidad, somos capaces de manejar sentimientos desagradables como tristeza y enfado | | | | 1 | 2 | 3 | 4 | 5 |
| 5- Durante situaciones adversas, estamos atentos a las necesidades de los miembros de nuestra comunidad | | | | 1 | 2 | 3 | 4 | 5 |
| 6- Nuestra comunidad es capaz de resolver situaciones de adversidad por sí sola, antes de recibir ayuda externa | | | | 1 | 2 | 3 | 4 | 5 |
| 7- Durante una adversidad, en nuestra comunidad nos organizamos antes de actuar, planificando lo que vamos a hacer. | | | | 1 | 2 | 3 | 4 | 5 |
| 8- Nuestra comunidad implementa y desarrolla habilidades necesarias para resolver sus adversidades y alcanzar sus metas | | | | 1 | 2 | 3 | 4 | 5 |
| 9- En nuestra comunidad no nos desanimamos ante la adversidad | | | | 1 | 2 | 3 | 4 | 5 |
| 10- Cuando hay problemas o adversidades, las personas de mi comunidad se unen y organizan para ayudar | | | | 1 | 2 | 3 | 4 | 5 |
| 11- Tenemos acceso a bienes y servicios del municipio (ciudad, comuna) durante situaciones de adversidad. | | | | 1 | 2 | 3 | 4 | 5 |
| 12- Durante una adversidad, las personas de nuestra comunidad controlan a aquellos que actúan mal o se desvían de las normas | | | | 1 | 2 | 3 | 4 | 5 |

*Note:* CR 12, original three-factor scale with 12 items: RE (items 4, 5, 9, 10), CS (items 2, 3, 11, 12), EC (items 1, 6, 7, 8). CR 9, three-factor scale with 9 items: RE (items 5, 9, 10), CS (items 2, 3, 11), EC (items 6, 7, 8).
